# Macrophage Plasticity in Cancer Therapy: Function, Timing, and Tradeoffs

**DOI:** 10.3390/ijms27156916

**Published:** 2026-08-01

**Authors:** Olga Kovaleva, Alexei Gratchev

**Affiliations:** 1 Institute of Experimental Oncology and Carcinogenesis, N.N. Blokhin National Medical Research Center of Oncology, Moscow 115478, Russia; ovkovaleva@gmail.com; 2 Center for Bio- and Medical Technologies, Skolkovo Institute of Science and Technology, Moscow 121205, Russia

**Keywords:** tumor-associated macrophages, macrophage plasticity, tumor microenvironment, immunotherapy, therapeutic window, myeloid reprogramming, cancer inflammation

## Abstract

Tumor-associated macrophages (TAMs) are considered to be one of the most attractive targets in cancer therapy but attempts to target them have produced variable and often contradictory results. Their phenotype and function are shaped by developmental origin, spatial niche, metabolic conditions, immune context, and treatment history. For this reason, TAM-directed therapy should not be approached as a simple problem of depletion or repolarization, but as a problem of function and timing. This framework integrates converging evidence from multiple independently reported studies of stage-, sequence-, and context-dependent macrophage targeting into a single decision structure, rather than proposing a new biological mechanism. We discuss here recurrent macrophage functions in solid tumors, including vascular support, matrix remodeling, immune exclusion, tumor-cell survival, antigen presentation, and tissue repair after therapy. We then consider how these functions affect the choice between depletion, recruitment blockade, reprogramming, and activation, and why the same intervention may be beneficial, ineffective, or harmful depending on lesion state and treatment sequence. Particular attention is given to therapeutic windows, biomarker-guided personalization, and adverse consequences of TAM intervention, including toxicity, compensatory myeloid substitution, loss of protective immune functions, repair-driven relapse, and the selection of inflammation-adapted or macrophage-resistant tumor cells. Effective TAM therapy will require functional precision, temporal precision, dynamic reassessment, and explicit attention to macrophage plasticity as both an opportunity and a source of risk.

## 1. Introduction

Tumor-associated macrophages are one of the most discussed targets in cancer therapy. This interest is well-justified, since macrophages affect angiogenesis, matrix remodeling, immune exclusion, treatment resistance, and tumor recovery after injury in many solid tumors [1,2]. At the same time, therapeutic targeting of tumor associated macrophages (TAMs) remains difficult. The main reason is that macrophages in tumors do not form one stable and uniform population. Their phenotype changes depending on tissue niche, developmental origin, metabolic stimulation, immune interactions, and treatment history [3,4]. Because of this, the same intervention may be useful in one situation and ineffective or even harmful in another.

In earlier work, macrophage biology in tumors was often discussed in the framework of M1 and M2 polarization. However, this model is too simplistic for the present stage of the field even if additional populations M2a, M2b and M2c are added. Studies of human macrophages have already shown that different signals induce partly overlapping but distinct programs, including matrix remodeling, scavenging, and secretory control, rather than one uniform alternative activation state [5,6]. Later single-cell and spatial studies extended this view to tumors and showed that TAM heterogeneity reflects ecological specialization within the tumor microenvironment [2,3]. For this reason, the main question is not whether macrophages are good or bad in general, but which macrophage functions are important in a certain lesion, when these functions become dominant, and whether they can be modified without losing useful myeloid support [2,7].

In this review, we consider macrophage-directed therapy as a problem of timing. We assume that the key therapeutic issue is not the macrophage itself, but the tumor-relevant function carried by a macrophage state in a particular situation. These functions may include vascular support, matrix remodeling, immune gating, trophic support, antigen presentation, or treatment-induced repair [8,9,10]. At the same time, macrophages can also preserve useful immune functions. Thus, broad depletion, untimely activation, or poorly chosen treatment sequence may remove beneficial myeloid programs together with harmful ones [11,12].

**Figure 1:**
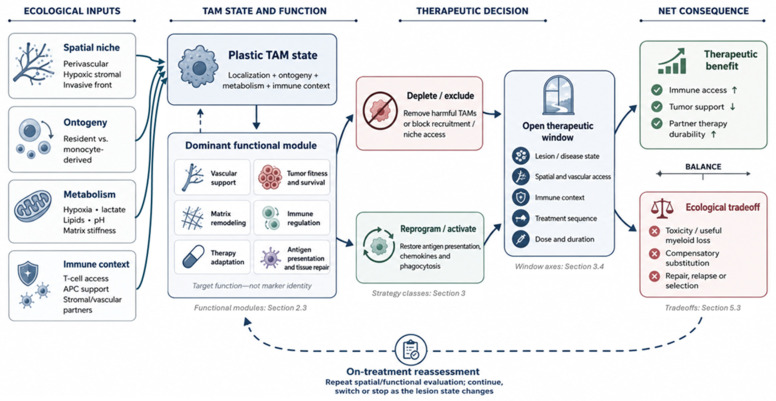
Proposed framework linking macrophage functional plasticity to therapeutic decision-making. TAM states are shaped by niche, ontogeny, metabolism, and immune context, which determine the dominant functional module (Section 2.3), the choice between depletion/exclusion and reprogramming/activation (Section 3) within an open therapeutic window (Section 3.4), and the balance between therapeutic benefit and ecological tradeoff (Section 5.3). The dashed arrow indicates the requirement for on-treatment reassessment.

This review is focused on TAMs in solid tumors. It does not aim to provide a catalog of all macrophage markers, all single-cell clusters, or all clinical trials. Instead, the emphasis is placed on several practical issues: how TAM states emerge, which functional modules are most relevant for intervention, when a therapeutic window is open, how the treatment decision may be personalized, and what tradeoffs follow macrophage perturbation (Figure 1).

## 2. Tumor Associated Macrophages (TAMs) States Emerge from Ecological Context

Having introduced macrophage-directed therapy as a problem of timing, we begin by asking how these macrophage states arise from the tumor’s ecological context. In different tumors, and even in different areas of the same tumor, macrophages may have different origin, localization, and function. Therefore, TAM biology is better understood as a spectrum of states shaped by ecological context than a polarization system [2,3]. We use “ecological” in the sense established for tumor biology generally, in which subclonal, stromal, and immune populations occupy distinct niches and compete under local selective pressure, rather than in its narrower sense restricted to relationships among free-living organisms [13].

### 2.1. Spatial Niche and Ontogeny Organize TAM Diversity

One of the most important factors that shape macrophage identity is spatial localization. In many tumor types, macrophages accumulate in reproducible anatomical niches such as perivascular areas, hypoxic and necrotic regions, fibrotic stroma, invasive fronts, and tertiary lymphoid structures [14,15,16]. These niches differ in oxygen tension, metabolite composition, matrix organization, stromal partners, and immune signals. It is therefore not surprising that macrophages occupying these niches acquire different phenotypes.

Perivascular macrophages are a clear example of this principle. TIE2 (tyrosine kinase with immunoglobulin and EGF homology domains 2)-expressing macrophages are localized near vessels, respond to ANG2 (angiopoietin 2), and promote angiogenesis and vascular permeability [8,17]. In contrast, hypoxic and necrotic tumor regions are often enriched with SPP1 (secreted phosphoprotein 1)-associated macrophage states linked to angiogenesis, matrix remodeling, and immune exclusion [18,19]. Stromal niches may contain macrophage populations involved in fibroblast interaction, matrix organization, and tissue maintenance [16,20]. Thus, localization is not only a descriptive feature of TAMs, but also a major determinant of their function.

Ontogeny adds another layer of complexity. Resident and monocyte-derived macrophages can remain distinguishable inside tumors and may occupy different spatial territories [21,22]. In glioma, resident microglia and blood-derived macrophages differ in both localization and transcriptional profile [23]. In breast cancer, resident-like FOLR2 (folate receptor beta)-positive macrophages are localized in perivascular stromal niches associated with CD8-positive T cell infiltration and favorable prognosis [20]. These observations indicate that macrophage identity in tumors reflects both the present tumor microenvironment and developmental history.

At the same time, TAM states are not completely random. Across many tumor types, single-cell studies repeatedly identify recurrent programs such as SPP1-associated, TREM2 (triggering receptor expressed on myeloid cells 2)- or APOC1-associated, interferon-responsive, and resident-like macrophages [2,3]. This means that plasticity is real, but it develops within a limited ecological framework.

### 2.2. Recruitment, Niche Signals, and Metabolism Shape Macrophage States

Macrophage states in tumors are formed by several interacting processes. First, myeloid cells have to be recruited and maintained in the lesion. Signals such as CSF1, IL34, CCL2, and CXCL12 regulate myeloid entry, positioning, and survival [1,24]. However, recruitment alone does not define function. After entering the tumor, macrophages are instructed by local stromal, vascular, immune, and metabolic signals.

One recurrent type of niche is the macrophage–fibroblast axis. In colorectal cancer, liver metastasis, and hepatocellular carcinoma, SPP1-positive macrophages interact with activated fibroblasts and thereby support collagen deposition, matrix remodeling, and immune exclusion [9,25,26]. A related role is played by endothelial niches, which support angiogenic macrophage programs through local growth factor signaling and direct cellular contact [27].

Metabolic conditions also make a major contribution to TAM specialization. Hypoxia, lactate accumulation, acidic pH, lipid handling, and matrix stiffness all influence macrophage phenotype [28,29,30]. Importantly, these signals do not simply generate one standard suppressive state. More often, they strengthen the local ecological program already imposed by niche structure. This probably explains why similar macrophage programs related to lipid metabolism, scavenging, fibrosis, or angiogenesis are found across different tumors despite strong tissue-specific variation [2,19].

Immune interactions provide another important layer of regulation. Macrophages communicate with T cells, dendritic cells, fibroblasts, and endothelial cells. Depending on the context, these interactions may support immune exclusion, adaptive resistance, or productive anti-tumor immunity [10,31]. Therefore, it is difficult to understand macrophage function if the surrounding immune circuit is ignored.

### 2.3. Recurrent Functional Modules of TAM Activity

Although TAM identities are heterogeneous, their tumor-relevant activities can be grouped into a smaller number of recurrent functional modules. This approach is more useful for therapy because it focuses on what macrophages do rather than the markers they express (Table 1).

These modules often overlap. For example, perivascular macrophages may simultaneously regulate angiogenesis, immune access, and post-treatment recurrence [8,36]. SPP1-associated macrophages often participate both in matrix remodeling and in immune exclusion [4,9]. Resident-like macrophages may support tissue integrity and local immune organization, but in other settings they may also contribute to tumor progression [20,37].

This distinction between identity and function is important for treatment design. In practical terms, the target is usually not a macrophage subset defined by a marker or marker combination, but a tumor-promoting function carried by macrophages in a specific ecological context.

## 3. Therapeutic Possibilities for TAMs

The previous section shows that TAMs are heterogeneous states shaped by tumor ecology. The next practical question is how this knowledge can be translated into therapy. In our view, TAM-directed treatment can be broadly divided into two main strategies: depletion or exclusion, and reprogramming or activation. These strategies are not equivalent. They are suitable for different tumor types and may fail through different mechanisms (Table 2).

### 3.1. Depletion and Exclusion

We begin with strategies that remove or exclude macrophages, before discussing strategies that redirect their function. The best developed depletion strategy is blockade of the CSF1-CSF1R axis. In preclinical studies, this approach reduced macrophage abundance, changed the phenotype of residual TAMs, and improved the effects of checkpoint therapy or radiotherapy [38,39]. However, even here, the mechanism is not simple removal. In pancreatic cancer, CSF1R blockade removed highly suppressive macrophages, but part of the therapeutic effect also came from the reprogramming of the remaining cells toward the antigen presentation and support of T cell function [39]. Thus, the boundary between depletion and reprogramming is often not strict.

Clinical studies confirmed that pathway involvement is possible, but anti-tumor activity was more modest than expected. Emactuzumab depleted CSF1R-positive macrophages in paired biopsies, but objective responses in monotherapy were absent and combination benefit was limited [40]. AMG 820 with pembrolizumab also showed target engagement but only modest clinical activity [41]. Pexidartinib plus durvalumab revealed another problem, since inhibition of FLT3-dependent dendritic-cell differentiation may weaken the expected synergy with checkpoint blockade [12]. These observations suggest that macrophage depletion itself should not be considered a sufficient endpoint.

Recruitment blockade has been studied particularly active in pancreatic cancer. CCR2 inhibition combined with FOLFIRINOX reduced inflammatory monocyte trafficking, decreased TAM content, increased CD8-positive T cell infiltration, and produced promising early signals [42]. However, later work showed that blocking one route of myeloid cells entry may induce substitution by another suppressive population rather than a durable effect [35]. Therefore, depletion or exclusion appears most rational in tumors that depend on continuous monocyte influx, in lesions with strong macrophage dominance, or in situations where a harmful niche has to be disrupted quickly [31,49].

### 3.2. Reprogramming and Activation

Reprogramming starts from another idea. It exploits the ability of macrophage to adapt and tries to direct macrophages toward immune support and tumor-cell killing.

One of the clearest examples is PI3Kγ inhibition. In checkpoint-resistant experimental tumors, this strategy did not require major macrophage depletion, but changed suppressive myeloid cells in inflammatory, MHC II-high, and T cell-supportive states [43]. CD11b agonists follow a similar principle. In pancreatic cancer, ADH-503 reduced the recruitment of suppressive myeloid cells and also reprogrammed the remaining TAMs toward antigen presentation and chemokine production [44].

Newer myeloid checkpoint targets are based on the same principle. In preclinical models, TREM2 targeting remodels myeloid cells away from suppressive states and improves the effect of anti-PD-1 (programmed cell death protein 1) therapy [45]. ILT4 blockade with MK-4830 offered early clinical evidence suggesting a myeloid resistance pathway could be therapeutically overcome [46]. Clever-1 blockade also belongs to this group, and bexmarilimab was associated with the activation of intratumoral immune programs and conversion of TAMs toward interferon-rich, antigen-presenting states in patients with disease control [47].

CD40 agonism lies between broad immune activation and macrophage reprogramming. In resectable pancreatic cancer, selicrelumab increased T cell infiltration, reduced fibrosis, decreased M2-like macrophages, and promoted dendritic-cell maturation [48]. However, studies combining selicrelumab with emactuzumab showed that biological activity is insufficient unless dose, schedule, and lesion are properly aligned [50].

### 3.3. Why Earlier Inflammatory Activation Often Underperformed

Earlier attempts to activate macrophages often produced disappointing results. This does not mean that macrophages cannot be activated. More often, activation was too systemic, too short, or insufficiently connected to adaptive immunity. In many cases, these strategies were also based on an oversimplified M2-to-M1 switch model.

TLR9 agonists illustrate this problem well. In phase III trials in non-small cell lung cancer, PF-3512676 added to chemotherapy did not improve outcome and increased toxicity [51]. Later approaches retained innate activation and tried to localize it. Intratumoral SD-101 with pembrolizumab improved local delivery and produced substantial responses in melanoma [52]. Vidutolimod represented a further refinement and showed durable responses in some patients with PD-1-refractory melanoma [53]. Still, lesion accessibility and tumor context remained important limitations.

STING agonists had similar difficulties. MIW815 combined with spartalizumab showed acceptable safety and expected cytokine induction, but objective responses were infrequent [54]. Therefore, the central question is no longer whether innate agonists can activate myeloid cells, but whether this activation is local, sustained, and functionally linked to antigen presentation and T cell recruitment.

Across CD40, TLR, and STING agonism, several practical constraints recur. Systemic myeloid activation carries a real hepatotoxicity risk: agonistic CD40 antibodies can trigger immune-mediated liver damage through tumor-conditioned hepatic myeloid cells, and this toxicity is schedule-dependent rather than fixed, since giving CD40 activation before or after chemotherapy determines whether it is lethal or tolerated [55,56,57]. Efficacy follows a similar logic: intratumoral or lesion-localized delivery (SD-101, vidutolimod) has outperformed systemic delivery (PF-3512676, MIW815), suggesting that the relevant therapeutic window is defined as much by where and how the agonist reaches myeloid cells as by the target itself [51,52,53,54]. That same localized-delivery requirement is itself a limitation, because intratumoral agonists depend on accessible, injectable lesions, which restricts their use in visceral or difficult-to-reach disease [52,53].

### 3.4. Decision Logic for Choosing a Strategy

The choice between depletion and reprogramming depends on tumor context. Depletion or recruitment blockade is more justified when macrophages are continuously replenished, numerically dominant, or driving a niche that must be dismantled quickly, such as therapy-induced rebound infiltration or stromal T cell exclusion [31,42]. Reprogramming is more rational when the macrophage compartment remains in place and still contains salvageable antigen-presenting or T cell-supporting functions [43,44,48].

In many tumors, none of the strategies are sufficient alone. Depletion may induce the compensatory accumulation of neutrophils, Treg cells, or resistant macrophage states [34,35]. Reprogramming may fail when the inflammatory signal is unstable, when T cells cannot enter the lesion, or when excessive stimulation causes counter-regulation instead of productive immunity [50,58]. Thus, what matters most is not combination as such, but biologically justified combination (Table 3).

### 3.5. What Has Improved and What Still Fails

The progress in TAM-directed therapy is not determined only by new molecular targets. It is also related to better study design. Drug delivery became more precise, combinations are selected more rationally, and patient selection is more often focused on myeloid-rich or macrophage-resistant tumors rather than unselected populations [47,61]. At the same time, endpoints are gradually improving. Instead of only measuring macrophage depletion or marker shifts, studies increasingly examine antigen presentation, T cell infiltration, matrix remodeling, and lesion control [12,61]. Table 4 places representative agents from each strategy side by side, with phase, efficacy signal, and principal limitation [12,35,58] to make these tradeoffs directly comparable.

However, the main causes of failure remain similar. Excessive dose may move the system outside the useful biological window. Off-target effects may negatively affect dendritic-cell support. Recruitment blockade may be bypassed by other suppressive myeloid cells. Innate activation may remain transient and not translate into stable adaptive immune control [12,35,58]. For this reason, TAM therapy should be viewed less as a list of drugs and more as a decision algorithm.

## 4. Window Concept for TAM Interventions

After the choice of strategy, the next important question is timing. The same macrophage-directed intervention may be helpful, neutral, or harmful depending on tumor stage and its relation to chemotherapy, radiotherapy, or immunotherapy. For this reason, TAM therapy should be considered not only as a drug-class problem, but also as a therapeutic-window problem.

Here we understand therapeutic window not simply as a period of time, but as a biologically defined state in which macrophage modulation is more likely to strengthen anti-tumor immunity than to remove useful myeloid functions or induce compensatory resistance. Table 5 shows four parameters that seem to be especially important here: tumor stage, vascular state and spatial accessibility, immune context, and treatment sequence relative to other modalities [33,66].

### 4.1. Timing Is the Missing Variable

One of the strongest arguments for the window concept comes from studies showing that TAM targeting has different effects in early and established tumors. In experimental models, anti-CSF1R therapy started in established tumors that depleted macrophages but did not produce sufficient tumor control. The same treatment started at tumor implantation had a stronger effect on tumor growth and more clearly improved the CD8-to-Treg ratio [33]. This does not mean that earlier treatment is always better. Rather, it indicates that macrophage dependence changes during tumor evolution.

This interpretation is consistent with the current knowledge of TAM dynamics. Time-resolved studies in pancreatic cancer showed that recruited monocytes first pass through an intermediate state and only later move into more stable terminal populations with different localization and functions [67]. In breast cancer, stabilin-1-positive macrophages were enriched in stage I and again in stage IV disease instead of increasing linearly with progression [68]. Thus, treatment planning should take into account whether the relevant macrophage program is emerging, established, or being rebuilt after prior therapy.

Radiotherapy in pancreatic cancer provides another example. In preinvasive lesions, irradiation accelerated progression and reduced survival while shifting macrophages toward a T cell-suppressive phenotype. In established invasive tumors, irradiation also promoted suppressive TAM polarization, but in this setting CSF1 blockade could partially restore anti-tumor immunity [76]. Therefore, the same influence may create a harmful window at one stage and a targetable window at another.

### 4.2. Vascular State and Spatial Accessibility Define Short Windows of Macrophage Dependence

Tumor stage is only one axis of timing. In many situations, the therapeutically relevant window is created by vascular changes and by the spatial localization of the key macrophage population.

After chemotherapy, some tumors enter a relapse-promoting phase characterized by the accumulation of perivascular MRC1-positive, TIE2-high, and CXCR4-high macrophages. These cells localize near vessels, produce VEGFA (vascular endothelial growth factor A), and support revascularization and regrowth [36]. In this situation, the important window is not simply “after chemotherapy,” but a more specific state defined by a CXCL12-rich vascular niche and close macrophage–vessel interaction. CXCR4 blockade during this interval delayed relapse [36].

Radiotherapy creates a related but distinct vascular window. Irradiation increases CSF1 production, induces delayed macrophage recruitment, and shifts these cells toward an immunosuppressive phenotype that limits the adaptive immune effect of radiation [69]. In glioblastoma and cervical cancer models, recurrence after irradiation also depends on SDF1 (stromal cell-derived factor 1)-CXCR4-driven myeloid re-entry into damaged vascular zones, and interference with this axis improves local control [70,71]. Therefore, one practical goal of TAM-directed therapy is to prevent tissue-repair programs from turning into tumor-repair programs.

Spatial accessibility is also important. In some tumors, the main problem is broad monocyte recruitment. In others, resistance depends on a small but critical perivascular or stromal macrophage population. This may explain why local or lesion-focused interventions sometimes give better effects than broad systemic activation when the decisive macrophage program is spatially restricted [53,54].

### 4.3. Immune Context Determines the Result of Macrophage Targeting

If vascular state defines where the window is located, immune context determines what macrophage targeting is likely to do inside that window.

In immune-excluded tumors, macrophages may act as a barrier preventing CD8-positive T cells from reaching tumor cells. Dynamic imaging in human lung tumors showed that stromal TAMs can retain T cells in the stroma and reduce their motility. CSF1R blockade restored T cell movement into tumor islets and increased the effect of anti-PD-1 therapy, although depletion alone had limited anti-tumor activity [31]. In this setting, the window is open when T cells are present but mislocalized.

Checkpoint sequencing studies support the same idea. CTLA-4 (cytotoxic T-lymphocyte-associated protein 4) blockade followed by, or combined with, CSF1R blockade was more effective than the reverse order, apparently because checkpoint therapy increased the abundance and vulnerability of suppressive CSF1R-positive myeloid cells [72]. The effect of radiation plus macrophage depletion also depended on the presence of an adaptive immune response and was enhanced by PD-L1 (programmed death-ligand 1) blockade [69]. Thus, macrophage targeting may work best as a second-step amplifier after antigen release or T cell priming has already started.

However, the opposite situation is also possible. Some treatment-induced macrophage states should not be removed but preserved or redirected. In ovarian cancer, neoadjuvant chemotherapy reduced markers of type 2 macrophage activation and induced a more inflammatory TAM phenotype. CSF1R inhibition shortly after chemotherapy worsened disease-free and overall survival, whereas later depletion had a much weaker effect [11]. Activated T cells can also recruit tumoricidal macrophages required for therapeutic efficacy [73], and selective targeting of CD206-positive macrophages disrupted a broader cDC1, NK-cell, and CD8 T cell axis in a recent study [74]. Thus, a macrophage-rich interval should not automatically be interpreted as a depletion window.

Another warning comes from lung cancer models in which antiangiogenic therapy plus PD-1 blockade worsened outcome. The problem was the formation of a macrophage-supported niche of PD-1-positive Treg cells that redirected checkpoint therapy away from CD8 T cells. Effective treatment required deeper remodeling of the macrophage compartment together with its regulatory partners [75]. Therefore, inflamed, immune-excluded, and immunosuppressive lesions should not be considered interchangeable therapeutic states.

### 4.4. Treatment Order, Intensity, and Duration Are Part of Mechanism

When the therapeutic window is defined biologically, treatment schedule becomes part of the mechanism rather than a technical detail.

Several studies show that an incorrect treatment order may remove benefit or induce toxicity. One of the clearest examples is agonistic CD40 antibody combined with chemotherapy. The administration of CD40 before gemcitabine caused CSF1R-dependent lethal hepatotoxicity, whereas gemcitabine followed by CD40 activation was tolerated [55]. This observation indicates that myeloid activation has to be timed according to the tissue state created by previous treatment.

Dose and duration are also important. In melanoma, combined anti-PD-1, anti-CD40, and anti-CSF1R therapy showed limited clinical activity. In the corresponding mouse models, a higher anti-CSF1R dose was less effective than a lower dose because it promoted the emergence of a suppressive macrophage population rather than stronger anti-tumor immunity [58]. Clinical data with pexidartinib plus durvalumab lead to a similar conclusion, because target engagement alone was not enough and off-target effects on FLT3-dependent dendritic-cell development may have reduced the intended immune effect [12]. Thus, stronger myeloid suppression does not necessarily mean stronger tumor control.

The practical implication is simple. If the window is narrow, the intervention should also be limited and functionally defined. It should be sufficient to disrupt the harmful macrophage program, but not so broad or prolonged that it removes antigen-presenting capacity, dendritic-cell support, or useful inflammatory macrophage states created by previous [11,58] treatment [11,58].

### 4.5. Windowed Intervention Requires Built-In Reassessment

**Figure 2:**
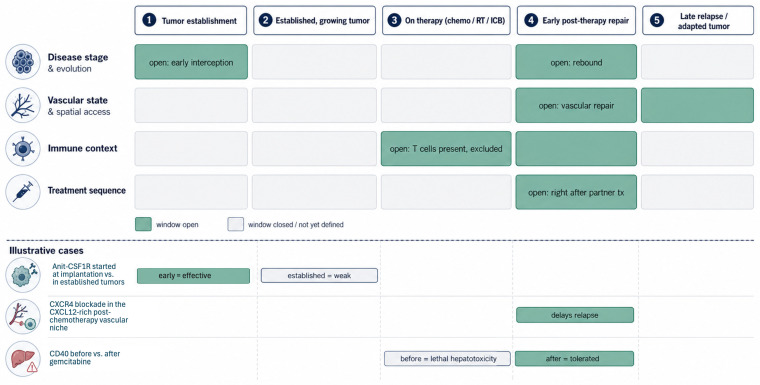
Temporal evolution of TAM states and therapeutic windows.

Since macrophage states change during treatment, window-based therapy cannot rely on fixed schedules alone. It requires reassessment (Figure 2).

At present, standardized stopping rules for TAM-directed therapy are not defined. However, available data support repeated biological evaluation rather than static treatment plans. Paired biopsies show that changes in macrophage phenotype, T cell infiltration, and matrix organization during therapy may differ substantially from baseline expectations [40,61]. New monitoring methods make such reassessment more realistic. In a mouse model, serial imaging with a CD163-directed nanobody tracer tracked TAM redistribution during immunotherapy and distinguished responding from nonresponding tumors by spatial pattern rather than total signal [77]. Repeated fine-needle aspirates in melanoma identified a therapy-resistant macrophage population emerging during BRAFi-MEKi treatment, which could not be reliably predicted from baseline samples alone [78].

For this reason, monitoring should focus not only on TAM abundance, but on whether the intended window is really opening or closing. In practical terms, this may include persistent T cell exclusion despite macrophage targeting, rebound myeloid recruitment, loss of dendritic-cell support, or toxicity indicating incorrect sequencing [12,31,55].

### 4.6. The Window Concept as the Organizing Principle for TAM Therapy

The main value of the therapeutic-window concept is that it turns TAM targeting from a list of agents into a framework for biological drug selection and timing.

Macrophage-directed intervention is more likely to work when it matches a transient lesion state. Such states may include early macrophage dependence during tumor establishment, myeloid rebound after radiotherapy, vascular repair after chemotherapy, immune exclusion with retained T cell potential, or myeloid compensation induced by checkpoint therapy (Figure 2) [31,33,36]. In contrast, TAM therapy is more likely to fail when it is used as continuous nonspecific pressure against a tumor ecosystem that has already adapted. It may also be harmful when it removes macrophage functions that became useful after earlier treatment, including antigen presentation, dendritic-cell support, or inflammatory macrophage states linked to treatment-induced immune activation [11,12].

Therefore, TAM therapy should be selected not only by target and not only by patient category, but also by lesion state, treatment sequence, and duration of intervention.

## 5. Personalization of TAM Interventions

After timing, the next practical issue is patient and lesion selection. Macrophage-directed therapy should not be personalized only according to the available drug class. A more useful approach is to identify the dominant macrophage-associated problem in a given tumor. This problem may involve stromal barrier formation, continuous suppressive recruitment, insufficient antigen presentation, vascular support, or the preservation of a myeloid compartment that can still be reprogrammed.

The goal is not to construct a very complicated omics classification for every patient. Rather, the aim is to define a minimal biomarker set that can guide the first therapeutic decision. In our view, three parameters are especially useful. First, immune contexture should be evaluated as a spatial continuum rather than reduced to a simple hot–cold classification [59,60]. Second, macrophages should be assessed not only by abundance, but also by localization, because stromal boundary, perivascular, hypoxic, tumor-nest-associated, and tertiary-lymphoid-structure-associated macrophages reflect different biological processes [16,20,31]. Third, functional state is more informative than broad M1-M2 terminology. Across tumor types, the CXCL9 (C-X-C motif chemokine ligand 9)-SPP1 axis separates immune-supportive macrophage programs from hypoxic, fibrotic, and exclusionary networks more effectively than classical polarization markers [2,4].

Therefore, personalization has two parts: baseline assignment and on-treatment reassessment. Baseline assignment defines the dominant macrophage problem before therapy. Reassessment determines whether this problem is changing in the expected direction.

### 5.1. A Practical Baseline Biomarker Set

We outline what such a panel should contain, then address why abundance alone is insufficient and how the resulting patterns map onto treatment decisions. A useful baseline panel should be focused on the dominant lesion ecology rather than on exhaustive cataloging of all myeloid subtypes. In routine stratification, the most informative markers are those that connect macrophage abundance with spatial position and functional state (Table 6).

This scheme is deliberately simple. It does not require whole-transcriptome or single-cell profiling for every case. For routine stratification, a limited panel that captures topology, niche, and function may be sufficient, whereas more detailed atlases are most useful when biology is ambiguous or when subgroup discovery is the goal [2,79].

### 5.2. Why Abundance Alone Is Not Enough

Even a lean biomarker set has to solve one central problem: total macrophage number alone is not enough.

Recent spatial studies show that macrophage-rich tumors are not one biological category. In colon cancer, spatially distinct macrophage populations have opposite prognostic associations. SPP1-positive macrophages in hypoxic and necrotic areas correlate with poor survival, whereas IL4I1 (interleukin 4 induced 1)-positive macrophages at the invasive front correlate with better outcome [16]. In breast cancer, FOLR2-positive tissue-resident macrophages occupy perivascular stromal niches, interact with CD8-positive T cells, and are associated with favorable survival [20]. A similar problem concerns markers often interpreted as uniformly suppressive. Stabilin-1 marks a distinct TAM subset rather than a generic macrophage state [68], and PD-L1-positive TAMs in breast cancer were enriched near T cells and associated with more immunostimulatory features, while PD-L1-negative TAMs localized closer to cancer cells and showed more suppressive and matrix-related programs [32]. Therefore, the meaning of a macrophage marker depends on both niche and function.

### 5.3. From Biomarker Pattern to Intervention Class

After abundance is placed back into spatial and functional context, biomarker patterns can be translated into a first therapeutic decision (Table 7).

This logic is still conceptual, but it is already practical. An immune-excluded, SPP1-rich, fibroblast-associated lesion does not present the same problem as a lesion with intraepithelial APC niches and insufficient activation. In the first case, the main task is barrier disruption. In the second case, it is preservation and amplification of antigen-presenting function.

The CXCL9-SPP1 axis is especially useful because it is low-dimensional but biologically informative. CXCL9-dominant tumors are associated with interferon signaling, dendritic cells, T cells, and better response to immunotherapy, whereas SPP1-dominant tumors are linked to hypoxia, angiogenesis, matrix remodeling, and immune exclusion [4]. Pan-cancer analyses support the same reduction, because macrophage states related to treatment response tend to fall into a limited number of recurrent functional roles within the tumor microenvironment rather than an unlimited number of unique subtypes [2,19].

At the same time, personalization should preserve useful macrophage niches, not only identify harmful ones. FOLR2-positive tissue-resident macrophages in breast cancer and intratumoral myeloid APC niches in ovarian cancer are good examples [10,20]. A lesion may therefore be macrophage-rich and still depend on macrophages for productive local immunity.

This axis should nonetheless be read as a low-dimensional summary of macrophage polarity rather than a validated clinical assay. It was derived largely from bulk and single-cell transcriptomic data rather than from a prospective biomarker trial, and tumor-type-specific recalibration appears to be necessary: independent pan-cancer and tumor-specific analyses have proposed adjusted ratios and cutoffs rather than reusing the original thresholds directly [80]. In practice, the axis is best used as one component of the panel in Table 6, alongside spatial and lymphoid-context markers, rather than as a stand-alone classifier.

### 5.4. On-Treatment Reassessment Is the Second Half of Personalization

Baseline assignment is only the first half of personalization, because the macrophage compartment is very sensitive to therapy.

Chemotherapy, radiotherapy, antiangiogenic therapy, and checkpoint blockade can all change macrophage localization and function. These changes may strengthen anti-tumor immunity, but they may also create new mechanisms of resistance [11,69,75]. Therefore, the same biomarker logic should also be applied during treatment, but for another purpose. The aim is not to define the initial problem again, but to determine whether the lesion is moving in the expected direction.

Direct observations support this approach. The same imaging and biopsy studies that motivate reassessment on the therapeutic-window axis apply directly here: TAM redistribution and resistance-associated macrophage populations are markers of whether personalization assumptions still hold [77,78]. In ovarian cancer, chemotherapy may induce myeloid networks associated with spatially localized CD8 T cell exhaustion [81], and in glioblastoma anti-CSF1R therapy, it may be followed by the development of a fibrotic recurrence niche not captured by macrophage counts alone [82].

In practical terms, baseline biomarkers should guide the first treatment step, whereas on-treatment biomarkers should determine whether therapy should be continued, intensified, changed, or stopped. Reassessment should focus on several direct questions: whether T cells entered the tumor, whether the macrophage program shifted from SPP1-like toward CXCL9-like, whether antigen-presenting capacity was preserved, and whether compensatory fibrosis or loss of dendritic-cell support appeared [4,12].

### 5.5. A Practical View of TAM Personalization

The value of personalization is not precision for its own sake. It is the avoidance of category errors.

Macrophage-targeted therapy fails when immune-excluded and immune-supported lesions are treated as the same state [31,59,60], when supportive resident macrophages are removed together with suppressive ones [20,83,84], or when a therapy-induced macrophage state is interpreted only from baseline tissue [77,78,81]. A minimal biomarker set can reduce these errors by asking four connected questions: where are the T cells, where are the macrophages, what are these macrophages doing, and how do these relations change during treatment.

This approach accepts the complexity of TAM biology without making patient selection dependent on very deep profiling in every case. For most translational settings, a hybrid strategy seems practical, with lean spatial pathology and small functional panels for baseline assignment and deeper profiling reserved for ambiguous lesions, resistance states, and biomarker discovery [2,79].

## 6. Adverse Effects and Tradeoffs of TAM Interventions

The previous sections considered TAM-directed therapy primarily as a problem of function, timing, and patient selection. However, the same framework also requires consideration of harm. Failure of TAM-targeted intervention may result not only from incorrect target selection, but also from the disruption of normal myeloid circuits. Such disruption may impair protective macrophage functions, damage normal tissues, promote expansion of alternative suppressive populations, or induce repair programs that subsequently support tumor recurrence [34,35,85,86,87,88,89]. For this reason, adverse consequences of TAM intervention are best viewed as ecological tradeoffs rather than as a single category of toxicity. This ecological framing follows a broader shift in the field away from treating macrophage depletion or activation as an endpoint in itself [89,90].

A useful distinction can be made between immediate and delayed tradeoffs. Immediate tradeoffs typically emerge during treatment or shortly after its initiation and include mechanism-based toxicity, the collateral loss of beneficial myeloid functions, the compensatory activation of alternative suppressive pathways, and situations in which target engagement occurs without meaningful ecological reorganization. Delayed tradeoffs become apparent later and more often reflect adaptive responses of the tissue ecosystem, including vascular or stromal repair, metastatic dissemination, rebound effects after treatment withdrawal, or the selection of tumor-promoting programs driven by inflammatory myeloid activity (Table 8).

### 6.1. Immediate Tradeoffs Include Toxicity, Loss of Protective Macrophages, and Compensatory Substitution

One immediate tradeoff of macrophage-directed therapy is mechanism-linked toxicity. This point is well-illustrated by agonistic CD40 therapy. In tumor-bearing mice, systemic anti-CD40 caused acute hepatitis with inflammatory infiltrates and parenchymal necrosis because tumor-conditioned hepatic myeloid cells were converted into ROS (reactive oxygen species)-producing effectors [56]. Later work showed that this toxicity was at least partly separable from efficacy, because TNF blockade reduced hepatotoxicity in CD40-based chemoimmunotherapy without abolishing the anti-tumor effect [57]. Thus, some myeloid-driven toxicities are not incidental, but arise directly from the intended immune perturbation.

CSF1R inhibition provides a more direct example for TAM-oriented therapy. Macrophage depletion disturbed extracellular-matrix homeostasis, increased MMP2 and MMP3 activity, and led to hyaluronan and proteoglycan deposition with clinically relevant edema [62]. In patients treated with emactuzumab, serum hyaluronan increased more strongly in those who developed edema [62]. Hepatic toxicity is another important warning for this drug class. In the ENLIVEN trial, pexidartinib produced mixed and cholestatic hepatotoxicity severe enough to stop enrollment early [63], and neoadjuvant testing in early breast cancer was later halted after vanishing bile duct syndrome [64]. These observations indicate that toxicity in macrophage-directed therapy may reflect on-target disruption of macrophage-dependent tissue homeostasis.

Another immediate tradeoff is the collateral loss of macrophage populations that restrain dissemination or support anti-tumor immunity. In metastatic models, CSF1R-dependent myeloid cells were required for NK-cell-mediated control of disseminated tumor cells because they trans-presented IL-15 and sustained NK-cell survival. Their depletion increased metastatic burden despite little effect on the primary tumor [83]. In breast cancer with high G-CSF (granulocyte colony-stimulating factor) signaling, anti-CSF1R treatment promoted an MHC II-low, mannose-receptor-high macrophage phenotype and enhanced lung metastasis [84]. Combined anti-G-CSF and anti-CSF1R treatment also depleted CD169-positive subcapsular sinus macrophages in lymph nodes and increased lymph-node metastasis [84]. Thus, the biological effect of macrophage depletion depends not only on what is removed from the tumor, but also on what is removed from protective immune compartments.

Compensatory substitution is a related and often rapid failure mode. In mammary tumor models, blockade of CSF1R or CSF1 increased spontaneous metastasis without changing primary tumor growth and was accompanied by increased serum G-CSF and the expansion of neutrophils in tumor, lung, and blood [85]. This effect was reduced by blocking G-CSFR [85]. A similar pattern was seen in monocyte-enriched glioblastoma. Genetic abrogation of monocyte chemoattractant proteins prevented monocyte infiltration, but neutrophils accumulated in compensation and drove hypoxia, necrosis, and proneural-to-mesenchymal transition through TNF signaling [92]. These studies show that suppression of one myeloid circuit may create another that is more harmful than the first.

A final immediate problem is the gap between pharmacodynamic activity and biological benefit. In recurrent glioblastoma, PLX3397 crossed the blood–tumor barrier, increased plasma CSF1, and reduced circulating CD14-dim, CD16-positive monocytes, yet objective responses were absent and six-month progression-free survival was only 8.8% [65]. A similar pattern was seen with pexidartinib plus paclitaxel, where plasma CSF1 rose markedly and circulating monocyte subsets fell, but toxicity remained substantial and efficacy was limited [91]. Therefore, systemic pharmacodynamic readouts can confirm that a drug is active without showing that the relevant tumor-supporting myeloid process has actually been disrupted.

### 6.2. Delayed Tradeoffs Often Arise Through Repair, Relapse, and Metastatic Support

Many longer-horizon failures are better understood as repair phenomena than as simple nonresponse. After treatment with the vascular-disrupting agent combretastatin A4 phosphate, tumors increased CXCL12 and rapidly recruited TIE2-expressing macrophages. Blocking this response increased necrosis and improved treatment efficacy [86]. After chemotherapy, Tie2 expression on infiltrating macrophages was likewise required for blood-vessel reconstruction and tumor regrowth [87]. These studies indicate that therapy-induced vascular injury can be converted into relapse through macrophage-dependent repair.

Repair-linked progression is not limited to angiogenesis. After paclitaxel, VEGFR3 (vascular endothelial growth factor receptor 3)-positive macrophages promoted lymphangiogenesis and metastasis through a cathepsin–heparanase–VEGF-C circuit, and the blockade of the VEGF-C-VEGFR3 axis reduced this effect [97]. Thus, treatment can educate macrophages into a state that supports dissemination even while the primary lesion initially regresses.

Some delayed risks become evident only after therapy is withdrawn. In breast cancer models, the cessation of CCL2 inhibition accelerated metastatic outgrowth by inducing monocyte influx, angiogenesis, and metastatic seeding [88]. This finding is important because it shows that transient suppression of a harmful pathway may be followed by an equally harmful rebound phase. In such settings, treatment discontinuation is not a neutral event.

Taken together, these observations suggest that macrophage perturbation can open a delayed repair phase that favors regrowth, vascular reconstruction, lymphatic remodeling, or dissemination rather than durable control.

### 6.3. Inflammatory Myeloid Pressure Can Interfere with Therapy and Reshape Tumor Evolution

Delayed harm may also arise when inflammatory myeloid activity itself becomes part of the resistance mechanism. Arlauckas and colleagues showed by intravital imaging that anti-PD-1 antibodies were rapidly removed from PD-1-positive CD8 T cells and captured by PD-1-negative tumor-associated macrophages through Fcγ–receptor interactions [93]. Blocking Fcγ receptors prolonged target occupancy on T cells and improved tumor regression [93]. In this setting, macrophages did not merely suppress immunity in a general way. They directly limited the activity of the partner immunotherapy.

Older experimental work suggested another type of delayed risk. Macrophage coculture increased the frequency of drug-resistant variants in a mouse mammary tumor line [98]. More recent in vivo work extended this logic by showing that myeloid-cell-derived ROS can drive epithelial mutagenesis, increase mutational burden, and accelerate invasive tumor evolution [94]. Repeated exposure of prostate cancer cells to cytotoxic macrophages likewise selected surviving populations with faster growth, greater clonogenicity, partial EMT-like change, and more aggressive xenograft behavior [95]. This strengthens the concern that inflammatory myeloid pressure may eliminate sensitive cells while enriching macrophage-resistant survivors. Conceptual work on paradoxical pro-tumor M1 macrophages argues that this risk should be considered explicitly when macrophage activation is prolonged or treated as an end in itself [96].

This issue is especially relevant for strategies that treat macrophage activation as inherently beneficial. In some settings, inflammatory macrophages may indeed support tumor control. In other settings, the same type of pressure may intensify genomic damage, favor resistant clones, or reshape the tumor into a more aggressive state. This is one of the central tradeoffs of macrophage plasticity in therapy.

The mechanistic basis of this tradeoff lies in the effector chemistry of activated macrophages. Reactive oxygen and nitrogen species, together with TNF, IL-6, and IFN-γ, are the same molecules that make inflammatory macrophages cytotoxic to tumor cells; at sublethal doses in surviving cells, they are also directly genotoxic, causing oxidative DNA lesions and mitochondrial DNA damage that raise mutational burden in adjacent epithelium [94,99]. This dual identity is consistent with the classical concept of cancer immunoediting, in which immune pressure not only eliminates susceptible tumor cells but also sculpts the surviving population toward escape variants [100,101]. Because macrophage activation is often chronic, epigenetically reinforced (‘trained’), and spatially diffuse rather than a single acute event, sustained inflammatory pressure may have more time to select for resistant or invasive clones than to eliminate the tumor outright [102]. This has direct practical implications for strategy choice: innate-agonist regimens that were systemic, prolonged, or poorly localized underperformed in the clinic, whereas localized, time-limited activation paired with adaptive immune engagement performed better [51,52,53,54]. The same logic argues against treating macrophage activation as a therapeutic endpoint in itself; its benefit depends on whether the resulting genotoxic and selective pressure is coupled to durable T cell-mediated clearance, or left to act alone on a tumor-cell population that can survive and adapt to it [95,96].

### 6.4. A Risk-Aware TAM Strategy Requires Dynamic Surveillance

These tradeoffs also change what should be monitored during treatment. Noninvasive imaging of CD206-positive macrophages detected relapse-prone tumors after chemotherapy before measurable regrowth and identified lymph-node metastasis earlier than tumor-cell imaging [103]. In patients, ferumoxytol-enhanced MRI correlated with TAM density in lymphoma and bone sarcoma samples, showing that macrophage-focused imaging can be translated beyond mouse models [104].

Other approaches capture different forms of harmful adaptation. Imaging of endogenous macrophage iron tracked response to CSF1R inhibition in breast cancer models [105], whereas multispectral fluorine-19 MRI revealed the spatial reorganization of macrophage populations during recurrence after radiotherapy [106]. These studies suggest that surveillance should not ask only whether macrophages are present, but whether they mark edema, compensation, repair, or an emerging resistant niche.

The main practical conclusion is that adverse effects of TAM intervention should not be separated from efficacy, because both arise from the same ecological process. A risk-aware strategy should therefore assess not only whether macrophages were depleted, shifted, or activated, but also which populations replaced them, which protective functions were lost, and whether the resulting myeloid state supports the restoration of normal tissue or the recovery of the tumor.

## 7. Conclusions

Having considered macrophage function, timing, personalization, and the tradeoffs of intervention in turn, we now draw these strands together.

TAM-directed therapy should not be considered as targeting of one cell population. In solid tumors, macrophages represent plastic states whose therapeutic significance is determined by localization, function, and treatment context. Therefore, the practical target is not the macrophage as such, but the macrophage-dependent process that maintains a particular lesion.

This shifts TAM therapy toward three connected questions: which macrophage function is dominant, when this function is vulnerable, and what will replace it after perturbation. Depletion, exclusion, reprogramming, and activation are useful only when they correspond to tumor ecology, treatment sequence, and the immune circuit that has to be restored.

The same logic applies to patient selection and risk assessment. Useful biomarkers should define spatial context, functional state, and the balance between harmful and protective myeloid programs. Equally important, macrophage targeting should be reassessed during treatment, because the same intervention may relieve immune suppression, remove beneficial myeloid support, induce compensatory repair, or select tumor cells better adapted to inflammatory or cytotoxic myeloid pressure.

Future progress in TAM therapy will likely depend not on a universal macrophage drug, but on macrophage modulation applied with functional precision, temporal precision, and the explicit control of ecological tradeoffs.

## Figures and Tables

**Table 1:** Targetable functional modules of TAM activity.

Functional Module	Principal Activities	Typical Consequences	Key References
Vascular support	Angiogenesis, vascular permeability, lymphangiogenesis	Tumor growth, dissemination, relapse after therapy	[8,17,27]
Tumor fitness and survival	Growth factor production, metabolic support, stress adaptation	Tumor persistence, treatment resistance	[1,24,28]
Matrix remodeling	Collagen turnover, fibroblast activation, niche formation	Invasion, desmoplasia, immune exclusion	[9,25,30]
Immune regulation	T cell exclusion, antigen presentation, checkpoint regulation	Immune suppression or immune support	[10,31,32]
Therapy adaptation	Recruitment of alternative suppressive cells, niche-dependent resistance	Relapse, compensation, treatment failure	[33,34,35]

**Table 2:** TAM-targeting therapeutic strategies.

Strategy	Main Rationale	Main Tools	Strengths	Main Liabilities	Key References
Depletion or exclusion	Remove dominant suppressive or trophic macrophages, or block their entry	CSF1 (colony-stimulating factor 1) and CSF1R (CSF1 receptor), CCR2 (C-C chemokine receptor type 2) and CCL2, CXCR4 (C-X-C chemokine receptor type 4), niche access pathways, selected cytotoxics	Fast debulking, clear pharmacodynamic readouts, useful when recruitment is continuous	Collateral loss of helpful myeloid cells, rebound recruitment, compensatory neutrophils or Tregs, schedule dependence	[38,39,40,41,42]
Reprogramming or activation	Convert retained macrophages into immune-supporting cells or release inhibitory myeloid checkpoints	CD40 agonists, TLR and NOD agonists, STING (stimulator of interferon genes) agonists, PI3Kγ (phosphoinositide 3-kinase gamma) inhibitors, CD11b agonists, TREM2, ILT4 (immunoglobulin-like transcript 4) and Clever-1 targeting, phagocytosis checkpoint blockade	Preserves useful myeloid scaffolds, often improves T cell recruitment and antigen presentation	Activity depends on delivery, dose, lesion accessibility, and intact lymphoid circuits; may remain incomplete without combination therapy	[43,44,45,46,47,48]

**Table 3:** Selection of therapeutic strategy.

Tumor Context	More Rational First Move	Why	Key References
Continuous monocyte influx and myeloid dominance	Depletion or recruitment blockade	Rapidly reduces supply-side pressure and may open a window for chemotherapy or checkpoint therapy	[31,42]
Retained but suppressive macrophages with salvageable T cell circuit	Reprogramming or checkpoint release on myeloid cells	Preserves antigen-presenting capacity and can restore chemokine production and phagocytic function	[43,44,48]
Fibrotic, matrix-rich, immune-excluded lesions	Neither alone	Requires combined attack on macrophages, stroma, and lymphoid access	[9,25,59,60]
Therapy-induced rebound or adaptive macrophage persistence	Sequence- or dose-optimized combination	Avoids rebound recruitment and state switching after initial perturbation	[33,36,55]

**Table 4:** Representative TAM-targeting agents in clinical development.

Agent	Target	Tumor Type(s)	Phase	Efficacy Signal	Major Toxicity	Key Limitation	Ref
Emactuzumab	CSF1R	Solid tumors; diffuse-type TGCT	I	No objective responses as monotherapy in solid tumors despite confirmed M2-like TAM depletion in paired biopsies	Edema linked to matrix-degrading protease/hyaluronan release	Target engagement without tumor regression in malignant disease	[40,62]
AMG 820 + pembrolizumab	CSF1R	CRC, pancreatic, NSCLC	Ib	Target engagement confirmed; only modest clinical activity	Manageable, class-consistent	Small, heavily pretreated cohorts	[41]
Pexidartinib	CSF1R	Recurrent GBM; TGCT (ENLIVEN); TNBC neoadjuvant; + durvalumab	II/III	GBM: no responses, 8.8% 6-month PFS. TGCT: ORR 39% (wk25), ~60% long-term—a non-malignant neoplasm. Breast: halted early	Hepatotoxicity, incl. vanishing bile duct syndrome	TGCT efficacy does not generalize to malignant tumors	[12,63,64,65]
CCR2i (PF-04136309) + FOLFIRINOX	CCR2/CCL2	Borderline resectable/locally advanced PDAC	Ib	49% objective response vs. 0/5 with chemo alone; resection in 71% (borderline) and 19% (locally advanced)	Tolerable in combination	Single-center, non-randomized, small cohort	[42]
Selicrelumab (neoadjuvant)	CD40	Resectable PDAC	I	82% T cell-enriched tumors vs. 37% untreated; fibrosis/M2-TAMs reduced; median OS 23.4 mo	Sequence-dependent hepatotoxicity with chemo (preclinical)	Small (*n* = 16), single-arm	[48,55]
Selicrelumab + emactuzumab	CD40+CSF1R	Advanced solid tumors	Ib	Biological activity present but insufficient without dose/schedule optimization	Combination toxicity constrains dosing	Two active myeloid agents did not simply add benefit	[50]
SD-101 + pembrolizumab	TLR9	Melanoma	Ib	ORR 78% (anti-PD-1-naive) vs. 15% (pretreated)	Injection-site reactions, flu-like symptoms	Benefit concentrated in checkpoint-naive patients	[52]
Vidutolimod + pembrolizumab	TLR9	PD-1-refractory melanoma	Ib	ORR 23.5%, median DOR 25.2 mo	Manageable, mostly local	Requires accessible/injectable lesions	[53]
MIW815 + spartalizumab	STING	Advanced solid tumors/lymphoma	Ib	Acceptable safety, expected cytokine induction, infrequent responses	Manageable	Systemic activation did not reliably yield response	[54]
MK-4830 ± pembrolizumab	ILT4	Advanced solid tumors	I	ORR 2% monotherapy vs. 24% combination	No DLTs, MTD not reached	Benefit appears to require checkpoint co-blockade	[46]
Bexmarilimab	Clever-1	Melanoma, gastric, HCC, ER + breast, biliary tract	I/II	Disease control 25–40% across tumor types; associated with improved survival	No DLTs across >100 patients	Benefit restricted to a DC-defined subset	[47]
Eganelisib + atezolizumab + nab-paclitaxel	PI3Kγ	1L metastatic TNBC	II	TAM reprogramming/ECM reorganization confirmed in paired biopsies; higher disease-control figures reported in conference/company disclosures pending full peer review	Manageable in combination	Mature peer-reviewed efficacy data still pending	[61]

**Table 5:** Therapeutic windows.

Window Axis	Window Opens When	More Rational TAM Move	Main Risk If Missed	Key References
Disease stage and evolution	Harmful macrophage states are still being established or have just been therapy-induced	Early interception or short reprogramming pulse	Established lesions may resist depletion or compensate	[33,67,68]
Vascular state and spatial access	Perivascular relapse niches or post-radiation vascular injury are active	Block recruitment or relapse-promoting vascular TAMs	Delay allows revascularization and regrowth	[36,69,70,71]
Immune context	T cells are present but excluded, or newly primed and suppressible	Remove gatekeeping TAMs or reprogram them to support T cells	Broad depletion can also disrupt useful APC or macrophage circuits	[31,72,73,74,75]
Treatment sequence	Partner therapy has created antigen release or a suppressive rebound that TAM targeting can exploit	Sequence TAM therapy after or with the right partner, not indiscriminately before it	Wrong order can erase benefit or produce toxicity	[12,55,58]

**Table 6:** Proposed minimal baseline biomarker panel for TAM-informed treatment stratification.

Biomarker Domain	What to Measure	Why It Matters	Practical Readout	Key References
Spatial immune contexture	Inflamed, excluded, or desert architecture, ideally quantified across regions rather than assigned as a binary label	Distinguishes absence of T cells from failed T cell access and from partially productive immunity	Haemotoxilin&Eosin plus CD8-centered multiplex IF or spatial pathology	[59,60]
TAM abundance and localization	Total macrophage burden plus stromal boundary, perivascular, hypoxic, tumor nest, or TLS-associated localization	The same macrophage burden means different things in different niches	CD68 or CD163 plus spatial markers such as panCK, CD31, fibroblast, or matrix markers	[16,20,31]
Functional macrophage program	CXCL9-to-SPP1 polarity, antigen-presentation features, suppressive pathways such as TREM2-, PI3Kg-, stabilin-1-, or IL4I1-linked states	Connects macrophage presence to what those macrophages are doing	Small RNA panel, multiplex IF, or limited targeted proteogenomic panel	[4,43,45,68]
Lymphoid support	Evidence of local APC support, CD28 costimulation, or productive macrophage-T cell proximity	Identifies lesions where reprogramming or APC support is more rational than depletion	Spatial proximity metrics, MHC II, CD74, CD80 or CD86, CD11c	[10]
Extension layer	Higher-resolution spatial profiling when baseline biology is ambiguous or unusually mixed	Refines subgroup assignment when the lean panel is insufficient	Spatial transcriptomics or deeper targeted profiling	[2,79]

**Table 7:** Translation of the biomarker patterns into a first-line therapeutic decision.

Biomarker Pattern	Biological Reading	More Rational First Move	Key References
Excluded tumor with stromal or boundary macrophage enrichment, SPP1-high or matrix-linked program, weak tumor-core CD8 access	Macrophages are part of a barrier niche with fibroblasts or matrix, limiting T cell entry	Depletion- or exclusion-disrupting strategies, often combined with checkpoint therapy and sometimes stromal targeting	[9,25,31]
Macrophage-rich lesion with retained T cells, MHC II or CD74 expression, CXCL9-leaning program, local APC niches	The myeloid compartment is suppressive only in part and may still support anti-tumor immunity if reprogrammed	Reprogramming or activation, myeloid checkpoint release, APC-supporting combinations rather than blanket depletion	[4,10]
TREM2-, PI3Kg-, or CSF1R-sensitive suppressive states with poor T cell function but salvageable immune circuitry	The dominant problem is macrophage-mediated suppression rather than complete immune desert	Selective depletion or reprogramming, usually in combination with PD-1 or PD-L1 blockade	[39,43,44,45]
Perivascular or relapse-linked macrophage niche with vascular-repair signatures	Myeloid cells are supporting treatment escape through vascular repair	Time-limited recruitment blockade or niche-specific interruption rather than prolonged broad depletion	[8,27,36]
Presence of supportive tissue-resident or APC-associated macrophages such as FOLR2-positive or PD-L1-positive immunostimulatory TAMs	Some macrophage populations are helping T cell recruitment or activation	Avoid indiscriminate macrophage depletion and favor selective editing, reprogramming, or combinations that preserve these niches	[20,32]

**Table 8:** Immediate and delayed tradeoffs of TAM-directed interventions.

Time Horizon	Main Tradeoff	Typical Mechanism	Key References
Immediate	On-target toxicity with partner therapy	Activated or depleted myeloid cells make normal tissue newly vulnerable	[56,57]
Immediate	Collateral loss of beneficial myeloid function	Broad CSF1R-dependent depletion removes cells needed for NK-cell or dendritic-cell support	[83,84]
Immediate	Apparent depletion with persistent harmful function	Sensitive macrophage subsets fall, but resistant vascular or suppressive subsets remain	[40,65,91]
Immediate	Compensatory substitution	Neutrophils, PMN-MDSCs, Tregs, or fibroblast programs fill the niche left by TAM targeting	[35,85,92]
Immediate	Overtreatment	Higher dose or poorly chosen sequence drives new suppressive states or severe toxicity	[12,58]
Delayed	Acquired resistance despite target engagement	Surviving macrophages are rewired by the microenvironment rather than eliminated	[78,82]
Delayed	Inflammatory or cytotoxic selection	Macrophage killing or inflammatory signaling enriches resistant or more aggressive tumor cells	[93,94,95,96]
Delayed	Therapy-linked inflammatory rewiring	Treatment enriches proinflammatory macrophages that support stemness or resistance	[11,81]
Delayed	Repair niches and withdrawal rebound	Vascular reconstruction, fibrosis, or renewed monocyte influx drive relapse	[36,86,87,88]

## Data Availability

No new data were created or analyzed in this study.
